# Kenya’s informal milk markets and the regulation–reality gap

**DOI:** 10.1111/dpr.12581

**Published:** 2022-03-01

**Authors:** Emma Blackmore, Alejandro Guarin, William Vorley, Silvia Alonso, Delia Grace

**Affiliations:** ^1^ IIED London UK; ^2^ ILRI Addis Ababa Ethiopia; ^3^ ILRI Nairobi Kenya

**Keywords:** dairy, food safety, food systems, governance, Kenya

## Abstract

**Motivation:**

Around 80% of milk in Kenya is marketed informally, providing livelihoods and contributing to the food security and nutrition of low‐income consumers. Government policy, however, is focused on formalization—primarily through licensing and pasteurization—with enforcement via fines, confiscation of milk, or closing the premises of informal actors.

**Purpose:**

This article seeks to better understand if, and why, Kenya’s informal milk sector and regulatory system are disconnected from one another and how the policy–reality gap might be better bridged.

**Methods and approach:**

To understand the nature and performance of Kenya’s informal milk markets and their governance, we used a mix of research methods and data sources, including surveys with informal market players, and key informant interviews with key sector stakeholders. Fieldwork was carried out in Nairobi in late 2018.

**Findings:**

Milk safety and quality matters to all actors in informal milk value chains. The trust‐based system used is effective in moderating behaviours and assessing and prioritizing quality and safety. Government policy is not accomplishing the stated goal of formalization: licensing levels remain low among informal actors. Pasteurization is not rewarded in the market. There is some evidence of suboptimal pasteurization processes being undertaken to satisfy regulators. There is a gap between the reality of Kenya’s informal milk sector and its regulatory system.

**Policy implications:**

The regulation–reality gap manifests itself as adversarial relationships between regulators and informal actors, and unnecessary transaction costs, missing opportunities for enhancing livelihoods, food safety, and food security. New approaches should build on and consider existing approaches taken by actors in informal food markets to ensure food safety and quality. Policy‐makers should seek to communicate more effectively with informal actors and engage in more constructive dialogue on inclusive ways forward.

## 1 INTRODUCTION

In many low‐ and lower‐middle‐income countries^1^ (L&LMICs), the market for milk and dairy is mostly informal. This means that it is characterized by small‐scale and traditional production, processing, and retail; and limited access to infrastructure including clean water, electricity, sanitation, and refrigeration. In informal markets, dairy products typically escape effective health and safety regulation, and operators are not licensed and/or registered, do not pay taxes, and receive little support from the public sector (Grace, 2015a).

Informal markets account for around 80% of all milk sold in Kenya. Most milk is sold raw (unpasteurized) and unpackaged, though some milk in informal markets may be sold after having been boiled and, increasingly, even pasteurized through small‐scale pasteurization units that are also informal. Informal milk markets are more popular than formal milk markets for several reasons: they are better able to provide food that meets sociocultural expectations regarding quality, they typically sell products at lower prices than formal markets or in smaller quantities that better suit the purchasing power of low‐income consumers, and they tend to pay higher prices to producers (Blackmore et al., 2015; Robinson & Yoshida, 2016).

Kenya’s dairy policy and regulation has been informed by Western ideas of progress and modernization. During colonial rule, partly in response to pressure from white settlers to control the sector and to limit the competition they faced from the indigenous population, the first piece of sector‐wide legislation (the Dairy Industries Act, 1958) was passed. The intention was to keep raw milk out of urban areas so that consumers would purchase pasteurized milk from the formal sector. The Act also established the Kenya Dairy Board (KDB) to “formulate the rules of the market and to monitor, sanction, enforce compliance and facilitate problem‐resolutions” (Atieno & Kanyinga, 2008). Concern regarding the health and safety of raw milk has been used to justify tighter regulations, which look remarkably similar to those passed in colonial times. The newly passed Dairy Industry Regulations (2021), permits raw milk to be sold only by producers direct to neighbouring consumers in rural areas, meaning only pasteurized milk should be sold in urban areas. The KDB states that the new regulations will “seek to address the issue of milk safety, boosting production capacity as well as addressing value chain and market challenges” (Omusolo, 2019).

The governance and regulation of informal dairy markets in Kenya is significant given that it is an important source of income and livelihoods, and a highly perishable commodity with serious public health implications. Though more recent data is unavailable, in 2004 it was estimated that raw milk sold through mobile vendors is estimated to provide 20 full‐time jobs per 1,000 litres of milk handled daily (SDP, 2004a). Milk bars—specialist outlets selling milk and other dairy products from fixed premises—offer 14 jobs per 1,000 litres of milk per day and employ large numbers of women. In comparison, the formal processing sector employs an average of 12.5 full‐time jobs per 1,000 litres of milk per day (SDP, 2004a). Even if formal jobs are more stable, wage rates between formal and informal dairy are broadly similar (SDP, 2004a).

Raw milk is highly perishable and susceptible to microbial growth and survival, and is a vehicle of food‐borne pathogens. In Kenya, milk‐related infectious diseases have been estimated to cause an annual loss of 53,000 healthy life years (Ndambi et al., 2018), also referred to as disability‐adjusted life years (DALYs) (Devleesschauwer et al., 2014). One of the main sources of microbial contamination in milk is handling equipment (Orregård, 2013). In Kenya, many informal milk vendors still use unhygienic plastic containers with small openings (jerry cans) for storing and transporting milk because of the high costs of purchasing the recommended aluminium or stainless‐steel containers (Alonso et al., 2018). Many lack refrigeration facilities or reliable electricity.

In addition to microbial contamination, residues from antimicrobial drugs used in veterinary treatment have also been found in milk, though these pose little direct risk to human health. The main public health risk of using antimicrobials in dairy animals is believed to be in fostering antibiotic‐resistant bacteria which directly or indirectly increase antibiotic resistance in human pathogens (Grace, 2015b), although the magnitude of this risk is yet as unknown. Other risks include toxins which cannot be eliminated by any form of heat treatment, including boiling, arising from certain strains of bacteria and to a much lesser extent aflatoxins from mycotoxin‐producing fungi which contaminate animal feeds before, during, and after harvest (Ahlberg et al., 2018). While recent evidence is lacking, a previous study in Kenya found that adulteration (with water, Blue Band margarine, or hydrogen peroxide) affected 5% and 10% of samples from consumer households and market agents, respectively (Omore et al., 2005)—though this was lower than the common public perception.

Microbial contamination and other health risks are not exclusive to informal dairy markets. Research carried out by SDP (2004b) in Kenya found that the bacterial quality of both raw and processed milk (pasteurized milk being sold through formal channels) was often quite low compared to national standards. An important determinant of milk safety in Kenya is not whether milk has been pasteurized or not, but the distance and length of the trading chain. Omore et al. (2005) found that most samples from short market chains and rural households in Kenya met the quality specifications for raw milk, but samples from long market chains and urban households did not. Similarly, over 60% of processed milk samples did not meet the specifications for pasteurized milk. Nyokabi (2021) collected milk samples from informal and informal value‐chain nodes and compared milk quality to the standards recommended by the Kenya Bureau of Standards. There were no differences in the quality of raw milk between locations or between nodes. Unsanitary milk‐handling practices were observed at farms and all value‐chain nodes. They argue that high levels of microbial contamination of milk pose a public health risk to consumers and show that urgent action is needed to improve milk quality.

Several theories have been offered as to why informality—and informal businesses in particular—exist and persist. These can broadly be categorized as theories of “*exclusion*” or theories of “*exit*.” Exit being that some of the informal self‐employed choose—or volunteer—to work informally to avoid registration and taxation (Chen, 2012), or due to stifling bureaucracy and over‐regulation; resistance or resentment towards government due to a perceived lack of procedural and redistributive justice and fairness (Williams et al., 2016). “Exclusion” describes the fact that some actors may be forced to operate in the informal economy out of necessity or tradition (Chen, 2012). There is growing acceptance among donors, researchers, and some policy‐makers, however, that the informal economy is not a discrete, self‐contained part of any economy, but rather is intrinsically linked to the formal economy (Chen, 2012), and many small enterprises pay taxes, fees, or bribes to local authorities (Robinson & Yoshida, 2016).

Informality poses significant challenges to policy‐making (Benson et al., 2014), in part due to its underexplored and under‐researched nature but also its association with negative outcomes. Informality is considered to undermine the performance of important parts of the economy (Kabwe et al., 2018). In the case of informal milk trade, policy‐makers’ concerns typically centre on food‐safety issues, as well as what is regarded as “unfair competition” posed by the informal sector to the formal sector, and are better able to avoid costs associated with taxes and licences.

The relationship between informal actors and governments can be very tense and defined by misunderstanding and conflicts. Brown and McGranahan (2016, p. 99) argue that “local authorities are inclined to view informal vendors and producers as illicit or even ‘illegal’, to the extent that their processes and arrangements do not conform to regulatory frameworks, and may interfere with the formal economy.” Informal vendors and producers are likely to perceive local authorities as a hindrance rather than a help because of the high levels of harassment and fines they face (Young & Crush, 2019). Harassment can take various forms, including the confiscation of goods or other property, threats of arrest, and the payment of bribes by informal actors to government officials. While governments may want to better regulate, control, and/or eliminate the informal economy, Roy (2005) states that informality must be understood not as the object of state regulation but rather as produced by the state itself, through the plans and legal apparatus it implements. A large disconnect can therefore exist between economic reality in many L&LMICs—and in informal sectors in particular—and regulatory systems.

This regulatory–reality gap matters for development. It misses opportunities for regulators to work with the reality of informal food systems to upgrade their performance, thereby maintaining food security and an important source of livelihoods for the many, and ultimately providing public goods. This article seeks to better understand if, and why, Kenya’s informal milk sector and regulatory system are disconnected from one another and how the policy–reality gap might be better bridged.

This research uses primary data obtained from fieldwork in Nairobi, Kenya’s capital, to understand the nature of Kenya’s informal milk markets; how trading relationships work; how value‐chain players perceive, and work to ensure, quality, and safety of the product they are selling or buying, including how consumers decide what to buy; and how the market is regulated. The research also documents the emergence of small‐scale “backstreet” pasteurizers in response to the government’s push for formalization.

The article is structured as follows: Section 2 outlines the methods used in the research, Section 3 presents the research results. Section 4 discusses the relevance of these findings in the context of the wider literature and Section 5 concludes by considering the possible implications of our findings for policy.

## 2 METHODS

To better understand the nature and performance of Kenya’s informal milk markets and their governance, we used a mix of research methods and data sources, including surveys with informal market players (producing both qualitative and quantitative data), and key informant interviews (KIIs) with key sector stakeholders, producing qualitative data. Existing literature was extensively reviewed to design the research tools and to summarize the state of play in the sector, to help understand the key knowledge gaps and to support some of the primary findings where necessary (see Blackmore et al., 2020). Fieldwork was carried out in late 2018.

The survey with value‐chain actors (producers, intermediaries, vendors, and consumers) was conducted in two peri‐urban locations in Nairobi: Dagoretti and Kasarani. Intermediaries are individual wholesalers, almost all of them men, who buy milk from producers to transport and sell it to vendors, sometimes via small‐scale pasteurization units. They transport it in small vans or motorcycles. The research locations were selected based on their importance as milk‐trading locations and ease of access. The sampling was purposive and was not statistically representative, but instead aimed to capture a diverse range of opinions and perspectives from market actors on milk demand and supply, considerations of health and safety, traders’ relationships with government (meaning intermediaries and vendors), business challenges faced, and how the market has changed in recent years. The total sample was 110, with the following breakdown: 41 vendors (37% of the sample), 42 consumers (38%), 17 intermediaries (15%) and 10 producers (9%). The survey used a mix of closed and open questions. Informed consent was obtained from all respondents, and permission obtained from local chiefs. All data was anonymized.

To recruit vendors (retailers, and specifically milk bars, shops, and mobile vendors) for the survey, we visited every vendor along known trading routes associated with milk‐vending outlets in informal settlements in the two study locations. We limited our survey respondents to the business owner rather than employees and skipped to the next vendor when the owner was unavailable or unwilling to participate. We excluded automated milk dispensers, as these supposedly sell pasteurized milk. Intermediaries, consumers and producers were recruited through either a direct approach in the field (e.g. the research team approaching consumers shopping from vendors we had surveyed, or intermediaries delivering milk to vendors), or through snowballing referrals (using contacts provided to us by vendors, or intermediaries).

In addition to the survey, we carried out 15 key KIIs with key stakeholders in the sector, including government (Ministry of Health; Public Health Office, Kenya Dairy Board; Kenya Bureau of Standards; Directorate of Livestock in the Ministry of Agriculture Livestock and Fisheries); private sector; donors; non‐governmental organizations; and civil society groups. These interviews were used to capture a range of opinions about the performance of the sector and the capacities and incentives of its actors to invest in better milk quality and safety and to comply with existing regulations. Respondents were assured that their names or affiliations would not be used to report results. After being transcribed, the interviews were coded by themes and analysed extracting and cross‐referencing the relevant text.

## 3 RESULTS

### 3.1 Kenya’s policy and regulatory environment

The government’s focus for economic transformation is on formalization, as evidenced by several national policies and plans, including the Big Four Agenda (launched in 2008, to be implemented until 2030) which has manufacturing and food security as two of its four priority areas (Government of Kenya, n.d.). For dairy, formalization means pasteurization. The specific strategy for the dairy sector is outlined by two key policy instruments. The first is the “new” Dairy Master Plan, which was completed and launched in 2012 to guide the development of the industry for the next 20 years. The plan sets a target for all milk to be pasteurized, to increase the percentage of milk marketed through formal channels, and to promote the marketing and consumption of packaged milk.

The second is the Dairy Development Policy of 2013, which recognizes that most milk is marketed in the informal sector and thus provides an appreciable employment opportunity, but raises concerns about its public health implications and states that formalization of the small enterprise sub‐sector in the dairy sector will be pursued. Measures for formalization include development and adoption of low‐cost technology for small‐scale dairy investors; investment in training programmes on safe milk handling; linkages with dairy industry stakeholders to improve the standards of milk processing in the informal sector; instituting public awareness campaigns on the dangers of drinking unprocessed whole milk and giving informal milk traders incentives for milk handling; and setting up a milk‐dealer certification system (MoALF, 2013).

Several laws regulate specific aspects of dairy production and trade. One of the key issues covered by regulation is the sale and handling of raw milk, though the regulations adopted in 2021 have made sales of raw milk direct from producer to consumer illegal, outside rural areas (see below). Before the 2021 Dairy Regulations was passed, the Public Health Act 2012 was perhaps the regulation most widely used by key regulators to govern the informal sector. It states that “certain milk is not to be sold if it is likely to have been contaminated or exposed to any infection or is in a condition likely or liable to prove unwholesome or injurious or dangerous to the health of man” (Cap 242, Section 134, p. 203). On this basis some authorities would not issue certain licences to those trading in raw milk. One interviewed government authority said that: “trade in raw milk is illegal under the Public Health Act. If you have licences and are selling raw milk you are formal but carrying out illegal practices.” Meanwhile, another government officer said that “as long as the trade operates within the confines of the law (i.e. have all necessary permits been obtained?) trade in raw milk is legal.”

The recently passed Dairy Industry Regulations of 2021 offer few concrete plans or opportunities for measures to allow for, or facilitate, value‐chain players to gradually move towards meeting the regulations. The regulations set the standards for dairy products and forbid the sale or processing of milk that has not undergone pasteurization, aseptic processing, retort‐sterilization, and refrigeration after pasteurization (Government of Kenya, 2021).

### 3.2 Licensing and enforcement

The tensions over the legality of raw milk signal a broader tension about the regulation of the informal dairy sector. Some government agencies would like to better support the informal sector but, as a government official argues, “it is currently very difficult to support them because they are so disparate. It is costly to work with them as individuals. We need better organization of vendors.” Other officials are more concerned with the need to regulate and enforce: “A number of challenges have emerged in the sector. For example, supermarkets now have dispensers. Anyone can enter the dairy business. And the dispensers [Milk ATMs] are still operating despite the KDB crack down. I have great concerns about the safety of these dispensers and how we regulate them.”

Some of the staff of government agencies that we interviewed argue that there are significant challenges involved in regulating the informal sector and enforcing existing regulations: “The dairy business is growing and changing and with it come new challenges every day. Outreach and coverage is hard, especially in the informal sector. This makes enforcement hard. In addition, the high turnover of traders in the informal milk market makes our work challenging.” Another official, working in an agency responsible for enforcing safety standards, expressed frustration at their lack of capacity and funding to carry out their work: “we need our own means of official transport so we can get around to do the checks and to have legitimacy with those we are conducting checks with. We need a means to keep the milk [collected for regulatory testing] cold, but we have limited portable containers and two carrying bags which is insufficient. Storage facilities are also inadequate when you get to the laboratories.” This person stated that the agency was unable to undertake any proactive, random sampling in those same markets.

Regulation and enforcement in the informal sector are further complicated by a complex licensing landscape. All actors who process, manufacture, prepare, or treat milk for sale, and distributors (intermediaries who buy for resale) are required to obtain a licence from the Kenya Dairy Board. Licensees pay a fee and a cess (levy) on the volumes of milk that they process or market. In addition to KDB licences, vendors must obtain licences from the Public Health Office and medical permits. These licences verify the safety of premises and food handling and the health of personnel working on the premises. A county‐level business permit (“annual single business permit”), the cost of which varies depending on the county, is also required. An additional Kenya Bureau of Standards licence is required if the business engages in value addition. Transporters of milk also have to obtain an additional licence (milk movement permit) from the KDB to operate as distributors (Blackmore et al., 2020).

Licences are an important, independent source of revenue for all licensing agencies. According to our interviews with multiple government agencies, devolution of power from central to local governments has increased the need for agencies to generate their own revenues, with licensing (and cess) being important sources. The establishment of a Kenya Food and Drugs Authority has been proposed to streamline licensing processes, but this has been met with significant resistance from other government agencies, which risk being dissolved or reduced in size if the Authority were to be established.

The level of compliance with licensing among informal vendors is uneven. Some government officials said in interviews that they believe the licensing rates to be high among small‐scale milk vendors: one mentioned that they estimated licensing rates of between 60% and 70%, while another mentioned rates of 90%. Traders were asked which licences they currently possess. Our survey data suggests that licensing levels are lower than government perceptions, but there are differences according to the type of licence and the value‐chain player (Table 1). There are also risks, despite reassurances of anonymity, that traders would be fearful of the possible repercussions of stating that they do not have licences. However, intermediaries’ stated compliance with licensing requirements are higher than vendors’ compliance—possibly due to the fact they often cross counties and are mobile, making them more likely to be subjected to random checks. In general, however, less than 50% of intermediaries or vendors report having the required licences, with the KDB licence being the least likely to be obtained by vendors.

**TABLE 1:** Licensing levels in informal traders (% of respondents having each license)

Licence/permit	Vendor (n=41)	Middleman (n=18)
City Council business permit	34%	28%
KDB licence	15%	44%
Public health certificate for food handlers	22%	56%

We found several reasons for traders not having a licence. Of those who offered reasons (25 vendors and 12 intermediaries), the most common was that it was too difficult to meet the requirements or standards associated with obtaining a particular licence, or that it was too costly. Vendors and intermediaries who did have the City Council permit or KDB licence reported paying an average of KES 5,620 (USD 52) per year for the permit and KES 6,920 (USD 64) per year for the KDB licence. Several vendors felt they were selling very small volumes of milk which did not justify paying the costs for licensing; they explained that their margins were too small to be able to absorb the costs: “I only handle 10 litres and I might make no profit if I pay for another permit.” Others believe that market trends of “reduced milk sales” justifies not having the licence. Others stated that they are new to the market so have not yet obtained the necessary licences, or they cannot afford the associated costs (4 vendors in each case). Licensing was also raised as a key business challenge by a number of other respondents, in particular the cost: “the costs of the council certificate and KDB licences are high”; “the licence price is high and there are plans to increase this” and worryingly, that: “sometimes licensing requires bribing” because vendors have been unable to meet the requirements associated with obtaining the licence.

Not having the licence leads to fear of harassment or losses for vendors (such as milk being confiscated or vendors having to temporarily shut down their businesses to avoid sanctions), bribes, and confiscation of milk for intermediaries. However, one intermediary mentioned that there is no point in obtaining a licence, since they will be harassed by authorities regardless.

Suggestions by vendors and intermediaries for improving the relationship with government include: the regulations being made less strict; value‐chain players being given more time to comply; better communication/dialogue between the authorities and value‐chain players; licensing requirements being streamlined and costs reduced.

### 3.3 Perceptions of traders and government about their relationship

The relationship between informal actors and regulators is fraught and characterized by mutual mistrust. Survey results revealed strong perceptions among vendors and intermediaries of an overwhelmingly negative attitude of government towards them. Almost half of interviewed vendors (19 of 41) and all intermediaries think the government has a negative view of them, which expresses itself as harassment (which may result in the need to pay bribes) or stringent regulations: requiring all milk to be pasteurized, or specific containers to be used.

Intermediaries have a particularly strong perception that KDB is extractive rather than supportive of them. They feel they are financially worse off because of the government. Similarly, about a third of vendors (14 respondents) stated that they experienced reduced income because of the government’s attitude towards them—for example as a result of confiscation of milk, having to pay bribes to avoid arrest, or having to close down their shops temporarily to avoid arrest. Several vendors mentioned paying fines of anywhere between KES 2,500 (USD 23) and KES 10,000 (USD 92), for not having the correct licences, not an insignificant amount for vendors from low‐income groups. Other financial losses include loss of customers, and therefore sales, when having to close to avoid detection during government inspections. Other vendors mention that they live in constant fear of being inspected, which they find psychologically draining.

A number of government agencies acknowledge the centrality of the informal sector in nutrition and livelihoods of poor people. Our interviews suggest that staff at national and regional government agencies recognize that the informal milk market “cannot be done away with,” but there are concerns about the public health risk often attributed to milk from the informal markets. One government official argued that “[raw milk markets] have been the preferred market because they are cheap and conveniently located. But the dangers of raw milk have not been adequately assessed by consumers. Chemicals are added, as are neutralisers to deter spoilage. Consumers buying raw milk have a perception that during processing something is taken away, or something is added. Based on our own independent testing, pasteurized milk is safe—apart from the odd example of malpractice on the processing side.” For this regulator, an unwillingness to license those trading in raw milk is justified on safety grounds.

Another interviewee from a government agency signalled a more pragmatic approach: “We recognize that milk is a very important foodstuff. It is so widely consumed. The cost of raw milk—which is half the price – is too important from a livelihoods and nutrition perspective. Despite outlawing them, 80% of the market is still made up of informal actors. But there are real safety issues…We need to find a better way to work with the sector…it is clearly here to stay.” Another officer argued that raw milk traders should be licensed so long as they meet safety standards, stating that “if a vendor does not meet standards (shown through milk testing), or does not have its licences, we can shut them down. However, this is not a regular occurrence. And we have not come across any serious issues in the [informal] sector yet.” It is important to bear in mind, as stated earlier, the significant capacity challenges that exist within government in terms of testing, however, despite this being a stated intention of the new Dairy Industry Regulations (Government of Kenya, 2021).

Many agencies, including the Public Health Office, the Directorate of Veterinary Services (under the Ministry of Agriculture, Livestock and Fisheries) and the Ministry of Health, perceive there to be lack of capacity within the informal sector to deliver safe and high‐quality milk. One agency official stated that “capacity and knowledge of milk handlers, those milking, obtaining the correct equipment for milking, ensuring good health of cows, knowledge of withdrawal periods after antibiotics (and incentives not to pour milk down the drain) is severely lacking.”

### 3.4 Informal market actors’ perceptions of milk safety and quality and practices to ensure it

Considerations of the quality and safety of milk shape trading relationships in the informal sector and are important decision‐making factors for trading partners. Informal traders (producers, intermediaries, and vendors) were asked to state why they choose particular suppliers or vendors. For about half of the vendors, milk quality was the most important reason. Other answers included cost, a sense of loyalty towards the supplier, favourable payment terms, and the ability to negotiate. For consumers, cleanliness was reported as the most important factor in determining where they buy their milk by around half of respondents. Several consumers also mentioned the importance of trusting specific vendors, and not experiencing problems previously associated with other vendors’ milk. For intermediaries, cost and convenience were the most important considerations.

Our survey reveals that trust and loyalty are crucial to trading relationships. Two thirds of producers, about half of intermediaries, and two thirds of vendors, stated that they never change suppliers, or do so rarely. Where there is a change in supplier, this is typically due to milk quality issues. For example, one intermediary explained that “if a farmer’s milk goes bad more than twice, I stop our agreement,” and another added that the trading relationship is over “if a farmer adds water to milk and if we disagree on who takes responsibility for the milk that spoils.” Consumers similarly stick to one or a very limited number of vendors, with almost all those surveyed stating that they do not shop around, suggesting high levels of trust. Respondents also attributed their loyalty to other factors, including the cleanliness of the shop, being friends with the vendor, quality, and price.

Perceptions of the meaning of milk quality and safety by vendors, intermediaries, and consumers were closely aligned (Table 2) (actors were able to give up to four responses). The dominant perception was that high‐quality milk means nothing has been added to it, it is fresh, and has a good thickness or consistency (which is linked to creaminess and butterfat content, and absence of adulteration). For these players, safe milk has a similar meaning. Producers differentiate slightly more between quality and safety, perceiving quality to be about the thickness and consistency of milk (based on its creaminess or butterfat content), and safety meaning nothing has been added (i.e. the milk is unadulterated) or milk is fresh.

**TABLE 2:** Meanings of quality and safety in milk to informal value‐chain players (number of mentions, by market actor)

	Consumers		Vendors		Intermediaries		Producers		Total
Definition of quality/safety	Safety	Quality	Safety	Quality	Safety	Quality	Safety	Quality	
Has nothing added to it	26	27	23	17	10	10	6	2	121
Is fresh	22	23	4	8	4	8	4		73
Good thickness/consistency	7	17	9	23	2	10		4	72
Has a “normal” colour	2	8	2	8					20
Comes from trusted vendor	6	3							9
Has been handled cleanly			5						5
Has been pasteurized			1		4				5
Other	1	3	4		7	9	7	5	36
**Total responses**	64	81	48	56	27	37	17	11	341

Formal testing for quality and safety—using equipment such as lactometers or alcohol tests—was reported by intermediaries only (15 out of 18 intermediaries used lactometers, and four used the ethanol or alcohol test). For those vendors who indicated they “test” milk, rudimentary measures such as using sight to check if the milk is the right colour or thickness, and smell or taste to check for freshness were reported. Two vendors explained that they use boiling to observe the consistency of the milk which gives an indication of whether anything has been added. None of the surveyed producers reported testing their milk before sale, and only two reported having had buyers test their milk (via a lactometer). Some vendors mentioned they found it difficult ascertaining whether milk was of good quality or not and whether it was likely to spoil. A key challenge in the sector remains access to, and knowledge of, testing equipment.

Producers, intermediaries, and vendors were asked to list up to three measures they take to ensure the quality and safety of milk before sale. The most commonly reported measure among producers was a quick sale (with five mentions), followed by electrical refrigeration (two mentions). Boiling was mentioned once, as was keeping milk close to the ground to keep it cool. The most frequently reported measure by intermediaries was pasteurization (six mentions) and a quick sale (six mentions), followed by electrical refrigeration (four mentions) and using separate containers for different batches of milk (not mixing morning and evening milk) (four mentions). The most common approach for vendors was electrical refrigeration (21 mentions), followed by boiling (20 mentions), a quick sale (10 mentions), and ensuring equipment for handling or storage is clean (10 mentions). Though not asked specifically what measures consumers take to ensure safety, all of the consumers stated that they boil milk before consumption.

Despite the lack of equipment for testing and the lack of widespread use of refrigeration reported by producers and intermediaries (and up to half the sample of vendors) along the chain, the perceived prevalence of spoilage was low. Most survey respondents in each value‐chain player group (producers–intermediaries–vendors) stated that in an average week less than 10% of their milk spoils, and many said they had no spoilage. However, spoilage was still cited as a significant business cost by a fifth of vendors—the second biggest after competition from other vendors. Adulteration and spoilage can disadvantage milk traders by tarnishing their reputation and reducing their sales—vendors state that consumers may not return to a vendor who they believe has sold them adulterated milk. Consumers confirm this, stating that if they believe they have become ill as a result of milk they can link back to a particular vendor, they will change vendor.

Even for those vendors who use refrigeration, power cuts can leave them vulnerable to spoilage; 10 vendors mentioned power cuts as a key challenge to maintaining the quality of their milk. Moving the milk quickly through the chain is used to manage the risk of spoilage. For those vendors who lack refrigeration facilities (around 50% of our sample), researchers observed that milk is left on the shop counter, with the aim being to sell it the same day. One vendor complained that if customers do not have money to buy milk at the right time, milk will not be purchased and consumed in time for it to avoid spoilage. In addition to quick sales, other measures among vendors include boiling the milk and cleaning equipment for handling or storage.

The survey highlighted consumers’ preference for raw milk from informal markets, which is then boiled at home before consumption. While the consumers surveyed assumed the milk they bought from vendors was raw, our surveys suggest that some intermediaries take milk to be pasteurized in small‐scale pasteurization units (see below) before sale to vendors, and some vendors boil milk to maintain freshness before sale to consumers. When consumers were asked why they prefer milk that (they believe) is raw, 50% (20 consumers) stated it was due to the milk’s “freshness,” followed by 20% (eight respondents) who said it was due to the taste and 18% (seven respondents) who said it was due to price. This is unsurprising, considering that formally pasteurized milk can be up to 60% more expensive than milk sold through informal markets. Most consumers reported paying KES 60 (USD 0.55) for milk purchased in informal markets, as compared to an average of KES 100 (USD 0.92) for pasteurized milk sold through formal channels in late 2018.

Several consumers feared pasteurized milk because of its associations with “chemicals” that allow it to have an extended shelf life. Milk from informal markets is drunk mainly by adults as mixed tea or “chai”, meaning it is boiled before consumption, followed by children under the age of five who drink plain boiled milk. Only nine consumers (out of 42) recalled experiencing any form of sickness—including vomiting or diarrhoea—as a result of drinking milk from informal markets; all of them linked the milk back to a particular vendor and changed the vendor they shopped from as a result.

### 3.5 Emergence of small‐scale processing units and associated increase in trading costs

Surveys with intermediaries and researcher observation during recruitment of value‐chain players provided evidence of a growth in small‐scale pasteurization units located in backstreets near producers and/or traders. Further research is needed to understand the scale of this trend. These are independent entities, not vertically integrated with a milk brand or formal processing company.

The surveys suggest that intermediaries are exposed to the most scrutiny from government authorities, largely because of the distances travelled, and have started to comply with the government push for them to only trade in pasteurized milk using these small‐scale processing units. A number of intermediaries surveyed collect milk from farmers, take it to a small‐scale plant to have it pasteurized for a fee, and then transport it onwards to vendors for sale. Intermediaries who work with these small‐scale processors suggest that the quality of pasteurization processes used in these small‐scale plants is inferior, for example because of machinery breakdowns, an insufficient number of heating and cooling cycles, or power cuts. As a result, some intermediaries think this option is not safer than raw milk.

In addition, the cost of pasteurization to the business is unrewarded by the market, since raw milk is readily available in the same retail outlets where intermediaries sell their pasteurized milk (i.e. typically informal settlements) which consumers prefer and typically buy at standard, and affordable, rates. Our survey suggests that consumers buying milk in informal markets are looking to purchase fresh, raw milk rather than pasteurized milk, so will not pay a premium for the latter. In order to comply with regulations, avoid harassment from authorities, and remain competitive, intermediaries must absorb the costs of pasteurization themselves. One intermediary stated that the business environment has become more challenging for him in recent years because he has suffered from “more harassment from KDB. They now require me to boil my milk [pasteurization], which costs me five shillings a litre and makes the business unprofitable.” Another intermediary trading in pasteurized milk explained that he is “squeezed from every angle—both suppliers and buyers. The competition is high. I am dealing with bigger volumes than before but actually making less money. We face competition from the big brands who are also selling pasteurized milk.”

There are also indirect costs associated with pasteurization, such as the time taken to wait for processing. One intermediary stated that: “KDB has pushed for pasteurization, which makes it more challenging from a time perspective, as we now have to wait for pasteurization. Often the machines break, and there will be a big queue of transporters waiting.” He explained that this can also leave the milk to spoil in the sun. A number of vendors who were linked to intermediaries who were trading in pasteurized milk seemed to be unsure about whether they were trading in raw milk or not, or insisted they were selling raw milk when their connection to the intermediaries would suggest otherwise. Intermediaries ultimately think they face a very difficult choice: consumer demand (for raw milk) and market dynamics, versus what the government wants (pasteurization).

## 4 DISCUSSION

Kenya’s approach to governing the informal sector has been very variable in recent years; ranging from ignore, to harass, to tolerate, to control (Blackmore et al., 2020). In the case of informal milk trade in Kenya, the case for regulation has been underpinned by food‐safety issues (though there is a lack of evidence on the incidence of milk‐borne disease in the country) driven by a modernization agenda, and by concerns about the informal sector posing “unfair competition” to the formal sector (Grace et al., 2007). However, government links to, and ownership of, major processors and the ability for government representatives operating on the ground to extract bribes from those who fail to comply with pasteurization or licensing requirements further incentivizes government to proceed with policies and practices that criminalize the informal sector. Nevertheless, there are differences between government agencies—with some stating support for maintaining and upgrading the informal sector. “Conflicting rationalities” (Watson, 2003) therefore exist not only between policy‐makers or urban planners and the communities they are planning for, but also within groups of policy‐makers.

However, recent approaches have, in practice, focused on controlling the informal sector, with harassment being one form of control via milk being confiscated or bribes being paid , or closing down their shops temporarily to avoid arrest. The adversarial relationships between regulators and informal actors lead to unnecessary transaction costs, undermining livelihoods. Informal actors perceive regulators to be predatory rather than supportive, and believe that they, and the sector, are worse off as a result of government.

Adversarial approaches to governance of the informal sector are all too common in L&LMICs (Kiaka et al., 2021; Patel et al., 2014; Resnick, 2017; Young & Crush, 2019). Robinson and Yoshida (2016) argue that the general orientation of food‐policy efforts is either to ignore the informal sector or to attempt to repress it. Many African countries still retain colonial‐era legislation on street vending that penalizes both sellers and buyers. Brown et al. (2010) found that in Ghana, Lesotho, Senegal, and Tanzania, municipal and public perception continues to see traders as untrustworthy, encroaching on territory designated for other uses, as being aggressive and dirty. Patel et al. (2014) found that more than a third of all street vendors interviewed in the Indian city of Madurai faced some form of harassment, including increased bribe payments, relocation, and threats of being shut down over the course of a year. Female street vendors were at particular risk of harassment. Young and Crush (2019) find that individuals engaging in informal economic activity are particularly vulnerable to abuse by authorities in the form of demands for bribes, harassment, threats, fines, the confiscation of goods, physical violence, and arrests. The fact that the state is often the source of these abuses shows the importance of governance reforms.

While heavy‐handed governance is often justified on the grounds of food safety, existing evidence suggests that these approaches may be counterproductive. Roesel and Grace (2014) find that draconian food‐safety policies that criminalize market actors could in fact make food safety worse. Similarly, research in Brazil suggests that frequent crackdowns reduce the incentives for those in the informal food economy to invest in the practices or equipment that would improve food safety (Azevedo & Bankuti, 2002). This is echoed by the findings of Grace et al. (2019) in Nigeria and Patel et al. (2014) in India, who found that rather than leading to better hygiene practices, the system of bribes, harassment, and forced closures acts as a disincentive for vendors to invest in better quality infrastructure and hygiene measures, and limits the quality of food. This is also highly likely to be the case in Kenya.

Food‐safety policies and interventions that do not take into consideration existing everyday consumption practices are unlikely to address acute food‐safety issues (Wertheim‐Heck et al., 2014). Our study suggests that trust and loyalty between buyers and sellers *do* help to maintain milk quality and safety in the informal market in Kenya. Other studies of informal food systems confirm the centrality of trust and social relationships in trade in the absence of effective regulatory and monitoring frameworks (i.e. policy environment) (Gerber et al., 2014). Trust and food safety are intertwined in the eyes of informal consumers. The situation of informal vegetable markets in Vietnam (Wertheim‐Heck et al., 2014) mirrors that of Kenya’s informal milk markets: food safety is either dealt with at home when cleaning and preparing foods, or is ensured through buying from a regular vendor. These vendors become “regular” because consumers have not experienced any issues of food safety when consuming food purchased from them. Consumers in Kenya’s informal markets appear to do both, with the overwhelming majority boiling milk before consumption, meaning that the presence of a hazard (e.g. pathogens or harmful substances) does not necessarily translate into significant risks to human health (Roesel & Grace, 2014). In India, 20% of consumers preferred eating food from a specific vendor, as they perceived products from “their” vendors to be better in terms of nutrition and quality than the same products of other street vendors (Patel et al., 2014). Consumers’ prioritization of cleanliness of both the vending site and the vendor themselves in our research in Kenya, is echoed by Rheinländer (2006) in Ghana, who found that—beyond price, availability, and accessibility—vendors and consumers are also highly concerned with neatness, which includes aspects of cleanliness, order, and aesthetic appearance.

In our research, producers and traders reported employing a variety of measures to try to ensure the quality and safety of milk. We did not test whether these approaches led to improved food‐safety outcomes, but evidence from other studies comparing formal and informal milk value chains in Kenya show no significant differences in quality or safety between chains (Nyokabi et al., 2021; Roesel & Grace, 2014). This is contrary to the perceptions among some government agencies that milk sold in informal markets is particularly dangerous for public health and that pasteurization is the answer to the multitude of food‐safety issues that arise in the production and trade of milk.

Kenya’s approach to governing informal milk markets may not only fail to improve on food safety, there may also be unintended consequences for food security. Despite the growth of modern retailing, informal food markets dominate the provision of food and drink to low‐income consumers. In a review of 23 studies on street food primarily from African cities, Steyn et al. (2014) found that street foods contributed significantly to the daily intake of protein, often at 50% of the recommended daily allowance. Skinner and Haysom (2016), found that the informal economy is a vital, if not the main, means by which the poor in South Africa attain a measure of food security. They argue that if policy approaches do not formally recognize the importance of the informal sector, the negative consequences will not only be shrinking employment and greater reliance on a resource‐poor state, but growing food insecurity (short‐ and long‐term), placing extra burdens on the state and society. Riley’s research in Malawi (2014) observed higher food insecurity among the poor when vendors were forced to the cities’ outskirts.

The results in our study point to opportunities for a more positive relationship between the government and informal milk‐market actors. Both regulators and market actors want to improve quality and safety, and the latter appear to be willing to comply with regulation, provided that it is more realistic. We found scant evidence of effective, sustainable, and scalable food‐safety interventions in informal markets, but there are some promising approaches. Building on the existing informal food system may be more successful than attempting to impose completely new systems. Resnick (2017), reviewed a variety of options for improving the governance of the informal economy beyond addressing food‐safety concerns, including institutionalizing regular engagement between local governments and informal workers within the management units of city councils and marketplaces—e.g. Zambia’s 2007 Markets and Bus Station Act (Government of Zambia, 2007). Dialogues and joint decision‐making of this kind encouraged many vendors to pay the requisite stall fees that cover investments in sanitation and other infrastructure, and fees are earmarked explicitly for improved infrastructure in markets, building trust between authorities and informal workers while also increasing local government revenue (Resnick, 2017). The International Livestock Research Institute (ILRI) has led training, certification, and licensing or marketing schemes and pilots to improve the health and safety practices of informal milk vendors with varying impact, though in some cases significant positive impact has been achieved for traders and food safety. However, these pilots have faced a number of challenges to scaling and sustaining (Blackmore et al., 2020), many of them political. At the very least, the Kenyan government should consider more meaningful dialogue and engagement with informal actors as a first step to improved governance.

There is a gap between economic reality and Kenya’s regulatory environment in Kenya’s milk sector. The economic reality is that low‐income consumers prefer raw milk and do not value pasteurization; producers prefer to sell into informal chains; consumers in informal markets are already taking approaches to try to ensure safe consumption; milk safety and quality is similar between informal and formal value chains; and informal traders struggle to navigate the licensing landscape. Policies, meanwhile, focus on pasteurization and licensing. The consequences of this void are transaction costs and adversarial relationships between government representatives and informal actors. Efforts by government to force the gap to close could be counterproductive for food safety, nutrition, and livelihoods.

An emergence of small‐scale pasteurizers shows some degree of compliance with regulations, but these sorts of innovations may fail to improve on food safety and may not be valued by low‐income consumers who are not concerned that the market as it currently stands does not offer pasteurized milk. And while the new regulations may force one aspect of formality to emerge— pasteurization—it may not drive a transition towards higher levels of registration or licensing or other aspects of formality. Conversely, the state may thus inadvertently maintain or increase levels of informality, as evidenced by Roy (2005) in other L&LMICs.

The policy environment incentivizes government agencies to maintain the complex licensing framework as a source of revenue generation and to fulfil plans for formalization under Vision 2030 by a performance‐related revenue‐raising model. Government links to major processors and the ability for government representatives operating on the ground to extract bribes further incentivizes criminalization of the informal sector. But serious questions remain over the government’s ability to meaningfully fulfil its plans for formalization. All government agencies are severely under‐resourced, limiting their ability to perform basic functions, such as random tests to ensure quality control.

Other approaches to governance are needed. The informal actors we surveyed felt dialogue with government agencies—specifically the sector regulator—could be improved. Informal vendors and intermediaries want government agencies to take a more pragmatic or positive approach to working with them, engaging them in dialogue and discussion as genuine stakeholders, and helping them to comply with relevant laws and standards in a more gradual manner. Informal actors need access to affordable finance to invest in simple equipment like lactometers and containers, and to be able to upgrade their premises. Government investment in market, water, and sanitation infrastructure is also critical to improving quality and safety throughout the chain. Inclusive and collaborative approaches to governance between policy‐makers and actors in the informal market might be better able to gradually upgrade milk quality and safety and to generate public revenue to facilitate sector reinvestment and upgrading.

## Data Availability

The data that support the findings of this study are available from the corresponding author upon reasonable request.

## Footnotes

^1^
See a classification here: https://datahelpdesk.worldbank.org/knowledgebase/articles/906519‐world‐bank‐country‐and‐lending‐groups
